# Surface α-Enolase Promotes Extracellular Matrix Degradation and Tumor Metastasis and Represents a New Therapeutic Target

**DOI:** 10.1371/journal.pone.0069354

**Published:** 2013-07-19

**Authors:** Kuan-Chung Hsiao, Neng-Yao Shih, Hsun-Lang Fang, Tze-Sing Huang, Ching-Chuan Kuo, Pei-Yi Chu, Yi-Mei Hung, Shao-Wen Chou, Yi-Yuan Yang, Gee-Chen Chang, Ko-Jiunn Liu

**Affiliations:** 1 Institute of Clinical Pharmacy and Pharmaceutical Sciences, National Cheng Kung University, Tainan, Taiwan; 2 National Institute of Cancer Research, National Health Research Institutes, Tainan, Taiwan; 3 Department of Cosmetology and Health Care, Min-Hwei College of Health Care Management, Tainan, Taiwan; 4 Department of Pathology, St. Martin De Porres Hospital, Chiayi, Taiwan; 5 School of Medical Laboratory Science and Biotechnology, Taipei Medical University, Taipei, Taiwan; 6 Faculty of Medicine, School of Medicine, National Yang-Ming University, Taipei, Taiwan; 7 Division of Chest Medicine, Department of Internal Medicine, Taichung Veterans General Hospital, Taichung, Taiwan; 8 Institute of Biomedical Sciences, National Chung-Hsing University, Taichung, Taiwan; 9 School of Medicine, China Medical University, Taichung, Taiwan; University of Patras, Greece

## Abstract

In previous research, we found α-enolase to be inversely correlated with progression-free and overall survival in lung cancer patients and detected α-enolase on the surface of lung cancer cells. Based on these findings, we hypothesized that surface α-enolase has a significant role in cancer metastasis and tested this hypothesis in the current study. We found that α-enolase was co-immunoprecipitated with urokinase-type plasminogen activator, urokinase-type plasminogen activator receptor, and plasminogen in lung cancer cells and interacted with these proteins in a cell-free dot blotting assay, which can be interrupted by α-enolase-specific antibody. α-Enolase in lung cancer cells co-localized with these proteins and was present at the site of pericellular degradation of extracellular matrix components. Treatment with antibody against α-enolase in vitro suppressed cell-associated plasminogen and matrix metalloproteinase activation, collagen and gelatin degradation, and cell invasion. Examination of the effect of treatment with shRNA plasmids revealed that down regulation of α-enolase decreases extracellular matrix degradation by and the invasion capacity of lung cancer cells. Adoptive transfer of α-enolase-specific antibody to mice resulted in accumulation of antibody in subcutaneous tumor and inhibited the formation of tumor metastasis in lung and bone. This study demonstrated that surface α-enolase promotes extracellular matrix degradation and invasion of cancer cells and that targeting surface α-enolase is a promising approach to suppress tumor metastasis.

## Introduction

Lung cancer is the leading cause of cancer-related death throughout the world [1]. Patients with advanced-stage lung cancer typically experience metastasis to multiple organs [2], [3], which are the major causes of death in these patients. Degradation of the extracellular matrix (ECM) by tumor cells is a critical step in tumor metastasis and multiple proteolytic enzymes are involved in this process, including plasmin, cathepsins, and members of the matrix metalloproteinase (MMP) family [4]–[6]. Plasmin has the ability to initiate MMP activation cascade by cleaving proMMP [7]. Blocking the activation of plasmin has been observed to prevent activation of MMP, and is thus a potential strategy in tumor therapy [8]. The process of cell-associated plasmin/plasminogen activation proceeds while plasminogen and a plasminogen activator, such as urokinase-type plasminogen activator (uPA), are recruited by macromolecular proteins or specific receptors such as uPA receptor (uPAR) that are expressed on the cell surface [9]. Plasminogen activation has been found to be positively correlated with tumor malignancy [10], and, in accordance, increased serum levels of uPA and uPAR have been positively correlated with poor prognosis in patients with prostate and breast cancer [11], [12]. These findings suggest that inhibiting or blocking the plasminogen/uPA pathways with a specific antibody (Ab) or inhibitor may interfere with the generation of plasmin, resulting in decreased pericellular proteolytic activity and tumor metastasis.

A highly conserved cytosolic enzyme in the glycolytic pathway, α-enolase (ENO1) has recently been reported to be involved in multiple functions [13]. ENO1 has been detected on the surface of pathogens and activated immune cells, where it serves as a plasminogen receptor and participates in systemic infection or tissue invasion and aids in the invasion of immune cells through blood vessels and tissues [14]–[16]. In our previous studies, we observed that ENO1 was overexpressed in the tumor of non-small cell lung carcinoma patients and displayed on the surface of lung cancer cells [17]. Elevated expression of ENO1 has been observed in many types of tumors [18], [19] and surface expression of ENO1 has been reported in lung, pancreatic and breast cancer cells [17], [20]–[22]. These observations suggest that surface ENO1 is likely to concentrate plasminogen on the cell surface, promote pericellular plasminogen activation, enhance ECM degradation, and increase invasion and metastasis of tumor cells [23]. However, direct evidence of the role of surface ENO1 in these processes in tumor cells is lacking.

To help fill this research gap, this study investigated the relationship and nature of interaction among ENO1, plasminogen, uPA, and uPAR; the effect of blocking ENO1 with Ab or silencing the expression of ENO1 with a specific shRNA plasmid in lung cancer cells; and the impact of adoptive transfer of Ab against ENO1 on the establishment of lung and bone metastasis by lung cancer cells in animal models. To our knowledge, this study is the first to directly investigate the role of surface ENO1 in ECM degradation and metastasis of cancer cells and the potential of Ab against ENO1 to inhibit or prevent tumor metastasis in lung cancer cells.

## Materials and Methods

### Mice and Cell Lines

All animal experimental protocols had been approved by the Institutional Animal Care and Use Committee of the National Health Research Institutes before study initiation. Male C57BL/6JNarl (C57BL/6) and NOD.CB17-*Prkdc^scid/^JNarl* (NOD-SCID) mice aged 5 to 6 weeks were purchased from the National Laboratory Animal Center, Taiwan, and maintained in the animal facility of the National Health Research Institutes. The murine LL/2 Lewis lung carcinoma and human A549 lung carcinoma cell lines transfected with the luciferase gene (LLC/luc and A549/luc) were purchased from Caliper Life Sciences (Alameda, CA). The LLC/luc cell line was cultured in DMEM with 5% FBS (Invitrogen, Carlsbad, CA) and the A549/luc was cultured in RPMI-1640 with 10% FBS. The human PE089 lung cancer cell line [17] was cultured in Minimum Essential Medium with 10% FBS.

### Ab

The mouse monoclonal Ab against mouse ENO1 (mENO1 Ab), the chicken polyclonal IgY against human ENO1 (ENO1 IgY) and the chicken control polyclonal IgY (control IgY) were produced as described previously [24], [25]. The mouse isotype-control Ab was obtained from Biolegend (San Diego, CA) and LTK BioLaboratories (Taoyuan, Taiwan). Rabbit anti-human ENO1 Ab and the corresponding isotype-control Ab were obtained from Genetex (San Antonio, TX). Mouse anti-human ENO1 Ab and the corresponding isotype-control Ab were obtained from Abnova (Walnut, CA). Ab specific for proteins of human and mouse origin were used according to the experimental design and origin of cells. Rabbit anti-uPA, anti-uPAR, and anti-plasminogen Ab (all of which react with proteins of human and mouse origin); normal rabbit IgG; mouse anti-ovalbumin (OVA) Ab; horseradish peroxidase (HRP)-conjugated goat anti-rabbit IgG and HRP-conjugated goat anti-mouse IgG were obtained from Santa Cruz Biotechnology (Santa Cruz, CA). PE-conjugated goat anti-mouse IgG Ab and FITC-conjugated goat anti-rabbit IgG Ab were obtained from Biolegend. Anti-focal adhesion kinase (FAK) Ab, HRP-conjugated goat anti-mouse light chain Ab and mouse anti-β-actin Ab were obtained from Millipore (Billerica, MA). Anti-α catenin Ab was obtained from Abcam (San Francisco, CA).

### Fluorescence-conjugated Ab Preparation

The mENO1 Ab and corresponding isotype Ab were labeled with Alexa Fluor 488 dye following the instruction of Alexa Fluor® 488 labeling kit (Molecular Probes, Eugene, OR) and purified with size exclusion purification resin.

### mENO1 Ag Preparation

Glutathione S-transferase (GST)-tag mENO1 was produced and purified as previously described [17]. The GST portion of the GST-tag mENO1 was removed using the Thrombin CleanCleave kit (Sigma-Aldrich, St Louis, MO) [26] and the resulting mENO1 was used to perform ELISA.

### mEON1 Ab ELISA

To detect the presence of Ab against mENO1 in mouse sera, 96-well plates were directly coated with 50 µl of mENO1 (6.5 µg/ml in phosphate-buffered saline) overnight at 4°C. The plates were then washed with phosphate-buffered saline containing 0.05% Tween 20 (PBST) 3 times and blocked with 3% bovine serum albumin (Sigma-Aldrich) in PBS (BSA/PBS) at room temperature for 1 hour (h). After blocking and washing, sera diluted in 1% BSA/PBS at a dilution of 1∶100 or a mENO1 Ab (as a standard concentration) were added to the wells for incubation at room temperature for 2 h. After washing, HRP-conjugated goat anti-mouse IgG (Jackson ImmunoResearch, West Grove, PA) diluted in 1% BSA/PBS at a dilution of 1∶10000 was added to all wells at room temperature for 1 h. After enzymatic activity had been initiated by incubation with 3,3′,5,5′-tetramethylbenzidine (Thermo Scientific, Rockford, IL) for 15 min at room temperature, the extent of the reaction was quantified by spectrophotometry (Spectra Max M5; Molecular Devices, Sunnyvale, CA) at an OD of 450 nm.

### Flow Cytometric Analysis

For examination of surface staining of ENO1, uPA, uPAR, and plasminogen in LLC/luc and PE089 lung cancer cells, 1×10^6^ cells were washed with PBS 3 times and then stained with 1 µg of the corresponding Ab at 4°C for 1 h. After washing with PBS, the cells were stained with PE-conjugated goat anti-mouse IgG or FITC-conjugated goat anti-rabbit IgG Ab at a dilution of 1∶200 at 4°C for 1 h. After further washing with PBS, the cells were suspended in PBS with 1% paraformaldehyde and analyzed by a flow cytometer (FACS Calibur; Becton Dickinson, Franklin Lakes, NJ).

### Immunofluorescence Microscopy

To detect the co-localization of ENO1, uPA, and uPAR, LLC/luc and PE089 cells were blocked with 1% BSA/PBS and then stained with the corresponding primary Ab diluted in 1% BSA/PBS at a dilution of 1∶1000 for 1 h at 4°C. After washing with PBS, the cells were further stained with fluorescence-conjugated secondary Ab diluted in 1% BSA/PBS at a dilution of 1∶200 for 1 h at 4°C. After fixing with 4% paraformaldehyde and counter staining with DAPI at a dilution of 1∶10000 (Sigma-Aldrich), cells were placed on a slide. The slide was immersed in Vectashield (Vector Laboratories, Burlingame, CA), mounted with a cover-glass, and sealed with nail varnish. The cells were then imaged using the ECLIPSE TE2000U laser scanning confocal microscope (Nikon, Melville, NY) and analyzed using EZ-C1 software (Nikon).

### Dot Blotting Assay

To detect the interaction among ENO1, uPA, uPAR, and plasminogen, 1 µg of each protein was blotted on a strip of nitrocellulose membrane and 1 µg of OVA (Sigma-Aldrich) was blotted as a negative control. After blocking with 5% BSA/PBS for 30 min at room temperature, the membranes were incubated separately with 1 µg/ml of each protein in PBS for 1 h at room temperature and then washed with PBS containing 0.1% Tween 20. After hybridization with the corresponding Ab (anti-ENO1, anti-uPA, anti-uPAR, or anti-plasminogen, respectively) in 1% BSA at a dilution of 1∶1000, the membranes were incubated with HRP-conjugated anti-rabbit or anti-mouse IgG Ab. The signals from the membranes were then visualized using the ECL system (Perkin-Elmer Life Sciences, Boston, MA).

### Duolink in situ Proximity Ligation Assay (PLA)

The Duolink in situ PLA (Olink Bioscience, Uppsala, Sweden) was performed following the manufactures’ protocol [27]. LLC/luc and PE089 cells were cultured on 1 cm×1 cm glass slips cm for 24 h, washed with PBS, and fixed with 3% paraformaldehyde for 10 min at room temperature. The fixed cells were again washed with PBS before blocking with a blocking solution (supplied in the Duolink kit) in a pre-warmed humid chamber for 30 min at 37°C. The cells were then incubated with primary Ab derived from 2 different species against the 2 proteins to be detected and incubated in a humidified chamber overnight at 4°C. Two corresponding secondary Ab against the 2 primary Ab (provided in the Duolink kit) and a connector oligonucleotide were then added. When the 2 proteins came into close proximity (<40 nm), the free ends of the 2 secondary Ab-attached oligonucleotides linked in a manner that allowed them to hybridize the connector oligonucleotide that precisely spans the gap between the 3′ and 5′ ends of the 2 Ab-tagged oligonucleotides. This process allowed the ends to be joined by enzymatic DNA ligation in the PLA ligation buffer, and the ligation products to be amplified by polymerase. After the polymerization reaction, fluorescence-labeled oligonucleotides complementary to the sequence on the circle were added for visualization of fluorescent signaling using a Leica TCS SP5 II laser scanning confocal microscope (Leica, Wetzlar, Germany) and quantification of PLA signaling using the DuoLink Image Tool v1 (Olink Bioscience).

### Invasion Assay

Cell invasion was examined using the Bio-coat Matrigel Invasion Assay System (Becton Dickinson) following the manufacturer’s protocol. Briefly, 1×10^4^ LLC/luc, PE089 or A549/luc cells were suspended in 0.5 ml of a serum-free medium containing either an ENO1-specific Ab or an isotype-control Ab (2 µg/ml Ab for LLC/luc and PE089 cells; 10 µg/ml IgY for A549 cells), and then added into transwell chambers consisting of matrigel-coated polycarbonate membranes with 8-µm pores. The transwell chambers were placed into wells containing medium with 5% FBS as a chemoattractant. After incubation for 24 h, the invading cells on the opposite side of the chambers were fixed with methanol and stained with Giemsa solution (Merck, Whitehouse Station, NJ) to allow for counting of the number of invading cells in the entire microscopy field under a stereoscopic microscope (Nikon).

### Pericellular Degradation Assay

To determine the correlation between ENO1 expression and ECM degradation by 3D matrigel invasion assay, the matrigel was diluted with PBS to a final concentration of 5 mg/ml. Fifty microliters of the diluted matrigel containing 25 µg/ml of fluorescence dye-quenched (DQ)-collagen (Molecular Probes), which was used as a substrate to determine the activity of pericellular ECM degradation by tumor cells, were then added into the wells of a Lab-Tek^tm^ chamber slide (Nunc, Rochester, NY). After the chamber slide had been incubated for 15 min at 37°C to allow solidification of the matrigel, 300 µl LLC/luc, PE089 or A549/luc cells at 1×10^5^ cell/ml were suspended in a matrigel containing 25 µg/ml of DQ-collagen, and then added into the wells and incubated for 30 min at 37°C for formation of a cell–matrigel mixture. After culture medium had been slowly added into the well on top of the settled cell–matrigel mixture, the cells were cultured for 5 days. The cultures were then fixed with 4% paraformaldehyde (300 µl/well) at room temperature for 10 min, after which the fixation was terminated by the addition of 300 µl of PBS–glycine solution (100 mM in PBS) for 10 min. After washing with PBS, 1 µg of ENO1-specific Ab (10 µg/ml in PBS) was added onto the slide and incubated for 1.5 h at 4°C. After washing, the slides were incubated with PE-conjugated goat anti-mouse Ab for mENO1-specific Ab or PE-conjugated goat anti-rabbit Ab for human ENO1-specific Ab at a dilution of 1∶200 for 1 h at 4°C, and the nuclei then counterstained with DAPI at a dilution of 1∶10000 for 5 min. After washing with PBS, the slides were immersed in Vectashield, mounted with a cover-glass, and sealed with nail varnish. The image of the 3D culture was observed using the ECLIPSE TE2000U laser scanning confocal microscope (Nikon) and analyzed using EZ-C1 software (Nikon).

To determine the effect of treatment with ENO1-specific Ab on the proteolytic activity, cells were cultured in a 24-well plate at 37°C for 1 day. The culture supernatant was replaced with a fresh serum-free medium containing mENO1 Ab, rabbit anti-ENO1 Ab or isotype-control Ab (2 µg/ml) and the cells were incubated at 37°C. At 30-min intervals, 100 µl of the culture supernatant was collected and incubated with 5 µg of soluble DQ-collagen for 30 min at 37°C. The fluorescence generated after the degradation of DQ-collagen was then detected using a Wallac Multilabel Counter 1420 (GMI, Inc., Ramsey, MN, USA).

### Plasmin and MMP Activity Assay

LLC/luc cells were cultured in a 24-well plate at 37°C for 1 day. The culture medium was replaced with a fresh serum-free medium containing 2 µg/ml of mENO1 Ab or isotype-control Ab and the cells were incubated at 37°C. At 30-min intervals, 100 µl of the culture supernatant was collected and incubated for 30 min at 37°C with H-Ala-Leu-Lys-AMC as a substrate for plasmin [28] (Anaspec, Fremont, CA) or Oregon Green® 488-conjugated gelatin (Molecular Probes) as a substrate for MMP2/9. The fluorescence was measured using a Spectra Max M5 spectrophotometer (Molecular Devices, Sunnyvale, CA).

### shRNA Transfection

All shRNA plasmids were purchased from the RNAi core of the Academia Sinica (Nankang, Taiwan). The target sequences of shRNA plasmid are 5′-ctggttagcaagaaagtgaat-3′ for mouse ENO1 (NM_023119); 5′-caacagccacaacgtctatat-3′ for control shRNA. The TurboFect Transfection Reagent (Fisher Scientific) was used for transfection of shRNA into cancer cells in accordance with the manufacturer’s instructions.

### Western Blotting Assay of ENO1

Cells were lysed in lysis buffer containing a protease-inhibitor cocktail (P3840; Sigma-Aldrich). The level of ENO1 in the cell lysates was analyzed with a Western blotting assay using mENO1 Ab, rabbit anti-ENO1, and mouse anti-β-actin Ab. Cytosol and membrane fractions from LLC/luc cells were obtained following the manufactures’ protocol of the Calbiochem® ProteoExtract® Native Membrane Protein Extraction kit (Millipore). FAK and α-catenin were used as loading controls of the cytosol and membrane fractions, respectively.

### Co-immunoprecipitation

Cells were lysed in lysis buffer and 50 µg of cell lysate was incubated with 2 µg of anti-plasminogen, anti-uPA, anti-uPAR or isotype-control Ab overnight at 4°C. The protein A/G Sepharose beads (Thermo Scientific) were then added into the mixture for 2 h at 4°C. The co-immunoprecipitated proteins were eluted and separated with SDS-PAGE, transferred to PVDF membranes, probed with mouse anti-human ENO1 Ab or mENO1 Ab and detected with HRP -conjugated goat anti-mouse light chain Ab.

### In vitro Proliferation Assay

The proliferation of cancer cells in vitro was determined by the MTT assay [29]. Lung cancer cells were suspended in complete culture medium and seeded onto a 24-well culture plate (Nunc) at a concentration of 5×10^3^ cells/well. After the culture plates had been incubated in a CO_2_ incubator for 0, 24, 48, and 72 h at 37°C, 50 µl of 3-(4,5-dimethyl thiazol-2-yl)-2,5-diphenyl tetrazolium bromide (MTT, 5 mg/ml; Sigma-Aldrich) was added into the wells. After the plates had been incubated in the CO_2_ incubator for an additional 2 h at 37°C, the culture medium was removed from all wells and 300 µl of DMSO (Sigma-Aldrich) was added into all wells. The plates were incubated at room temperature for 30 min in the dark before the absorbance of each well at an OD 540 nm was measured using the Spectra Max M5 spectrophotometer.

### Lung Metastasis Animal Model with i.v. Implantation of Tumor Cells

LLC/luc cells (1×10^5^) were injected i.v. into C57BL/6 mice on day 0. The mENO1 Ab or an isotype-control Ab (400 µg/mouse) was administered by i.v. injection 2 h before tumor implantation. Thereafter, Ab (100 µg/mouse) was administered by i.v. injection every 2 days during the experimental period. The lung metastasis of LLC/luc cells was imaged by using the IVIS™ live-imaging system (IVIS Lumina II, PerkinElmer, Santa Clara, CA) [30]. The lung tissue was then embedded in paraffin and cut into 5-µm-thick sections for H&E staining. Alternatively, A549/luc cells (1×10^5^) were injected i.v. into NOD-SCID mice on day 0. ENO1 IgY or control IgY (2 mg/mouse) was administered by i.v. injection 2 h before tumor implantation. Thereafter, 500 µg of ENO1 IgY or control IgY were administered by i.v. injection to each mouse every 2 days during the experimental period. The establishment of lung metastasis was detected by the IVIS system.

### Lung metastasis Animal Model with s.c. Implantation of Tumor Cells

LLC/luc cells were s.c. injected into the right flanks of C57BL/6 mice. After detection of lung metastasis, LLC/luc cells in the lung were isolated and cultured in regular medium, and 1×10^5^ cells of the resulting cells (LLC/luc SC LM1^st^) were s.c. injected into the right flank of each mouse on day 0. Treatment with mENO1 Ab was performed as described above. Growth of LLC/luc cells was monitored every 2 days and the tumor volume estimated using the modified ellipsoid formula (π/6×[length×width^2^]) [31]. The tumor was irradiated for 3 Gy with a RS2000 X-ray Irradiator (Rad Source, Suwanee, GA) on day 18. The presence of lung metastasis was determined using the IVIS system. For the detection of Ab absorption in tumor, 1×10^6^ LLC/luc cells were injected s.c. into C57BL/6 mice. After tumor volume had reached 200 mm^3^, 100 µg Alexa Fluor 488-labeled mENO1 Ab or isotype-control Ab was injected i.v. into mice. The fluorescent and luminescent intensity were detected 4 h later by the IVIS system.

### Bone Metastasis Animal Model

LLC/luc cells were injected into the left cardiac ventricle of C57BL/6 mice as previously described [32], and establishment of bone metastasis subsequently imaged using the IVIS system. After LLC/luc cells in the long bone of the hind limb had been isolated and cultured in regular medium, the resulting LLC/luc BM1^st^ cells were intracardially injected into the mice to obtain the second generation of LLC/luc bone metastatic cells (LLC/luc BM2^nd^). The LLC/luc BM2^nd^ cells were injected into C57BL/6 mice to study the effect of administration of mENO1 Ab on bone metastasis using the treatment protocol described above. The tibia and femur were removed and embedded in paraffin for preparation of 5-µm-thick sections. Subjection of the sections to H&E staining confirmed the presence of tumor in the bones [33].

### Statistical Analysis

All numerical data are presented as mean±SD values. The data were analyzed by performing the student’s *t* test or Log rank test using GraphPad Prism 4.0 sofware (GraphPad Software, Inc., La Jolla, CA). A *P* value of ≤0.05 was considered statistically significant. *p<0.05, **p<0.01 and ***p<0.001.

## Results

### ENO1 Interacts with uPA, uPAR and Plasminogen

**Figure 1:**
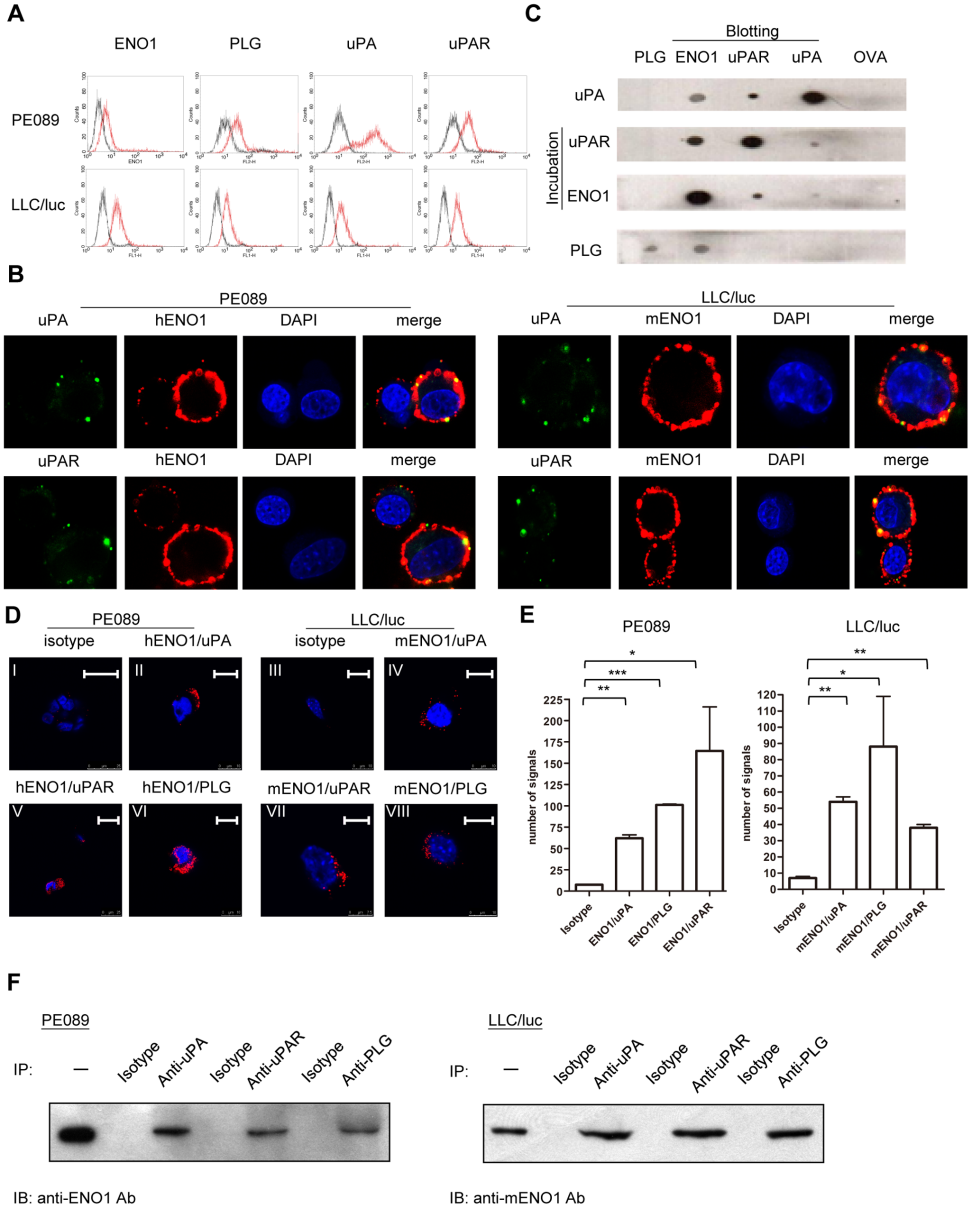
Expression of and interaction among ENO1, plasminogen, uPA, and uPAR. (A) PE089 and LLC/luc lung cancer cell lines were stained with isotype-control Ab (dark lines) or Ab against ENO1, plasminogen (PLG), uPA, and uPAR (red lines) and analyzed by flow cytometry. (B) PE089 and LLC/luc ells were stained with Ab against ENO1 (red fluorescence), uPA, and uPAR (green fluorescence) and analyzed under a confocal microscope. Cells were counterstained with DAPI (blue) for observation of the nucleus. (C) After dotting with PLG, ENO1, uPAR, uPA, and OVA, each strip of the membrane was incubated with uPA, uPAR, ENO1, or PLG, washed, and then hybridized with an Ab against uPA, uPAR, ENO1, or PLG, respectively. The bound Ab was detected using the ECL system. (D) Cells were incubated with ENO1-specific Ab together with Ab against uPA (ENO1/uPA), uPAR (ENO1/uPAR), or PLG (ENO1/PLG). The proximity of 2 bound Ab on the cells was detected using the PLA system. A positive amplified signal (red dots) from the PLA system indicated that 2 detected proteins/Ab were co-located within 40 nm of each other. Cells were counterstained with DAPI (blue) for observation of the nucleus. Isotype: control Ab corresponding to the 2 primary Ab used for ENO1, uPA, uPAR, or PLG. Scale bar (upper right corner): 7.5 µm (VII), 10 µm (II-IV, VI, VIII), and 25 µm (I, V). (E) The number of signal dots from (d) was quantified using the DuoLink Image Tool v1 software. The error bars were defined as mean±SD. *p<0.05, **p<0.01 and ***p<0.001. (F) Protein lysates from PE089 (left) and LLC/luc (right) cells were immunoprecipitated [53] with anti-uPA, anti-uPAR, anti-PLG and corresponding isotype-control Ab. The total cell lysate and co-immuoprecipitated proteins were then immunoblotted [20] with Ab against human (left) and mouse ENO1 (right). -: cell lysate without IP.

The expressions of ENO1, uPA, uPAR and plasminogen on the surface of PE089 cells, a human lung cancer cell line [17], and the murine Lewis lung carcinoma cells transfected with the luciferase gene (LLC/luc) were examined and confirmed by FACS analysis (**Figure 1A**). Similar results were observed in other human lung cancer cell lines and the parental murine LLC cells (**Figure S1**). Under confocal microscopy, uPA and uPAR were observed to co-localize with ENO1 on the cell surface (**Figure 1B**). To directly examine the ability of these molecules to interact, we performed a cell-free dot blotting assay and found that ENO1, uPA, and uPAR interact with each other, while plasminogen only interacts with ENO1 (**Figure 1C**). No reactivity of a control protein ovalbumin with these 4 proteins was observed. The Durolink in situ PLA was used to further examine the nature of this interaction in cells. Detection of a positive amplified signal from the PLA indicates that 2 detected proteins are located within 40 nm of each other [27]. As shown in **Figures 1D and 1E**, ENO1 was found to be located closely with uPA, uPAR, and plasminogen in both LLC/luc and PE089 cells. In consistent with these findings, ENO1 was demonstrated to be co-immunoprecipitated with plasminogen, uPA and uPAR in LLC/luc and PE089 cells (**Figure 1F** **).**

### ENO1 Accumulates at the Invading Front of Cancer Cells and ENO1 Blockade Reduces ECM Degradation and Cell Invasion

**Figure 2:**
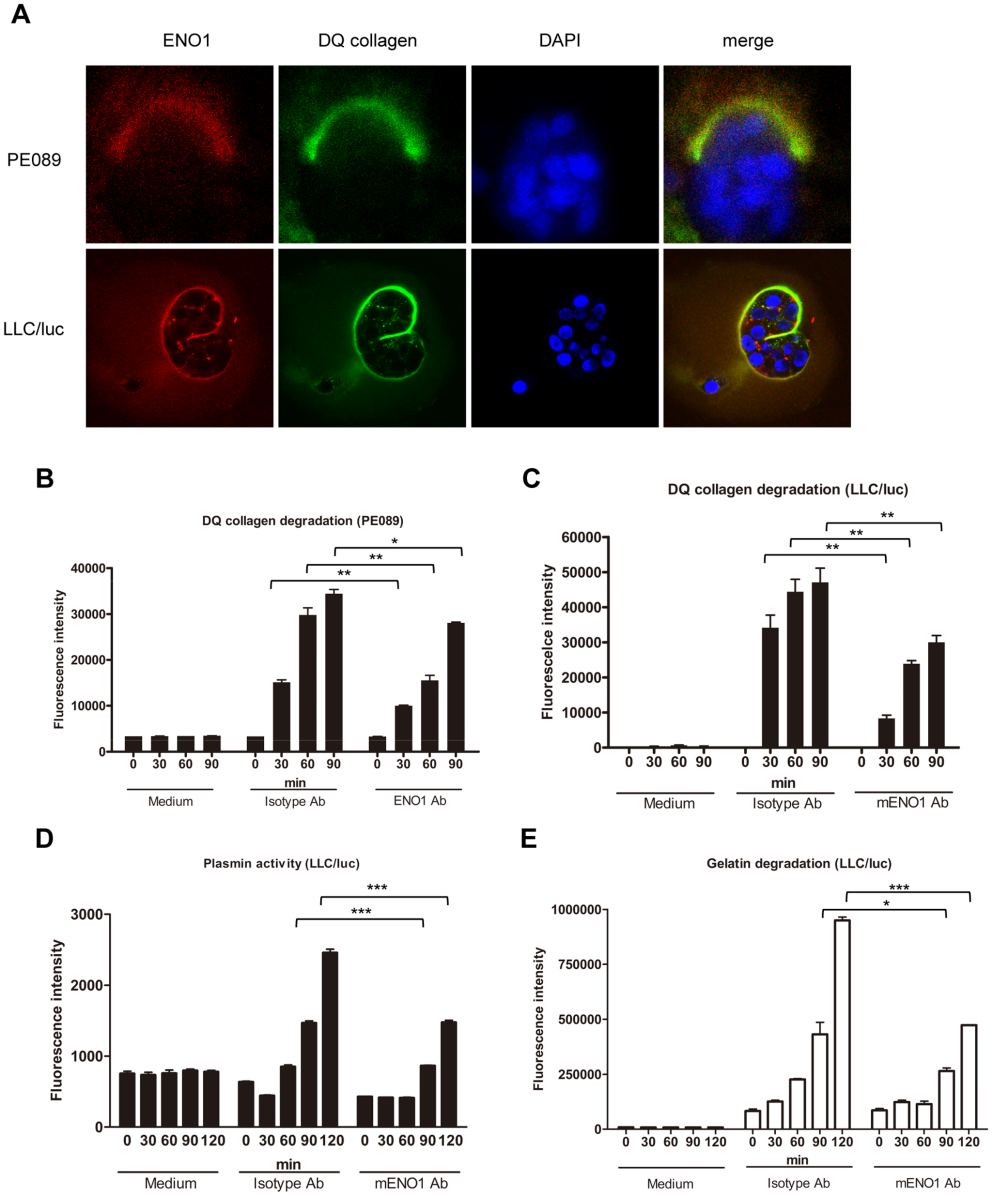
ENO1 accumulated at the pericellular proteolytic site and Ab against ENO1 suppressed collagen degradation and protease activities. (A) PE089 and LLC/luc cells were embedded in matrigel containing fluorescent DQ-collagen and then further stained with a PE-labeled Ab against ENO1 to detect the presence of ENO1. The cleaved product of DQ-collagen (green fluorescence) and the presence of ENO1 (red fluorescence) was observed under a confocal microscope. Cells were counterstained with DAPI for examination of the nucleus. (B and C) PE089 and LLC/luc cells were cultured in the presence of Ab against human and mouse ENO1 or isotype-control Ab (2 µg/ml). The culture supernatant was subsequently collected at 30-min intervals and mixed with soluble DQ-collagen. The fluorescence generated after the degradation of DQ-collagen was measured. (D and E) LLC/luc cells were cultured in medium containing mENO1-specific or isotype-control Ab (2 µg/ml) at 37°C. At 30-min intervals, 100 µl of the culture supernatant was collected and incubated with H-Ala-Leu-Lys-AMC (D) or fluorescence-labeled gelatin (E) as a substrate for plasmin or MMP2/9, respectively. The fluorescence released from the degraded substrates was then measured. *p<0.05, **p<0.01 and ***p<0.001. The error bars were defined as mean±SD.

To examine the nature of the correlation between ENO1 and ECM degradation, PE089 and LLC/luc cells were embedded in the matrigel containing DQ-collagen, which emits green fluorescent signals during degradation, to allow the growth of cells in a 3-dimension (3D) fashion, and then further stained with a PE-labeled Ab against ENO1 to detect the presence of ENO1. As shown in **Figure 2A**, the degraded products of DQ-collagen (green fluorescence) and ENO1 (red fluorescence) were observed to be co-localized at the interface between tumor cells and the matrigel, suggesting that ENO1 is associated with collagen degradation and accumulates at the invading front of tumor cells in a 3D culture system.

To directly investigate the role of ENO1 in degradation of ECM components, LLC/luc and PE089 cells were cultured in the presence of Ab against mouse and human ENO1 or an isotype-control Ab. The culture supernatant was subsequently collected at 30-minute (min) intervals and mixed with soluble DQ-collagen for detection of the fluorescence generated after the degradation of DQ-collagen. While the supernatant in the tumor cell culture containing the isotype-control Ab was found to promote time-dependent degradation of DQ-collagen, the supernatant in the tumor cell culture containing the ENO-specific Ab was found to be significantly inhibited from promoting this process (**Figures 2B and 2C**). Because the medium alone failed to mediate this time-dependent degradation of DQ-collagen, the above results indicate that blockage of surface ENO1 reduces the proteolytic activity derived from these tumor cells. We next examined the effect of incubation with ENO1-specific Ab on the activation of plasmin in LLC/luc lung cancer cells. The time-dependent increase of plasmin activity was found to be significantly inhibited in the culture supernatant of cells treated with ENO1-specific Ab (**Figure 2D**). Since surface ENO1 binds plasminogen, it is likely that inhibition of cell-associated plasmin activity by ENO1-specific Ab may be due to interference on the binding of plasminogen. To test this possibility, we performed a dot-blotting assay and found that binding of soluble plasminogen, uPA and uPAR to ENO1 dotted on the membrane was indeed reduced in the presence of ENO1-specific Ab (**Figure S2**).

**Figure 3:**
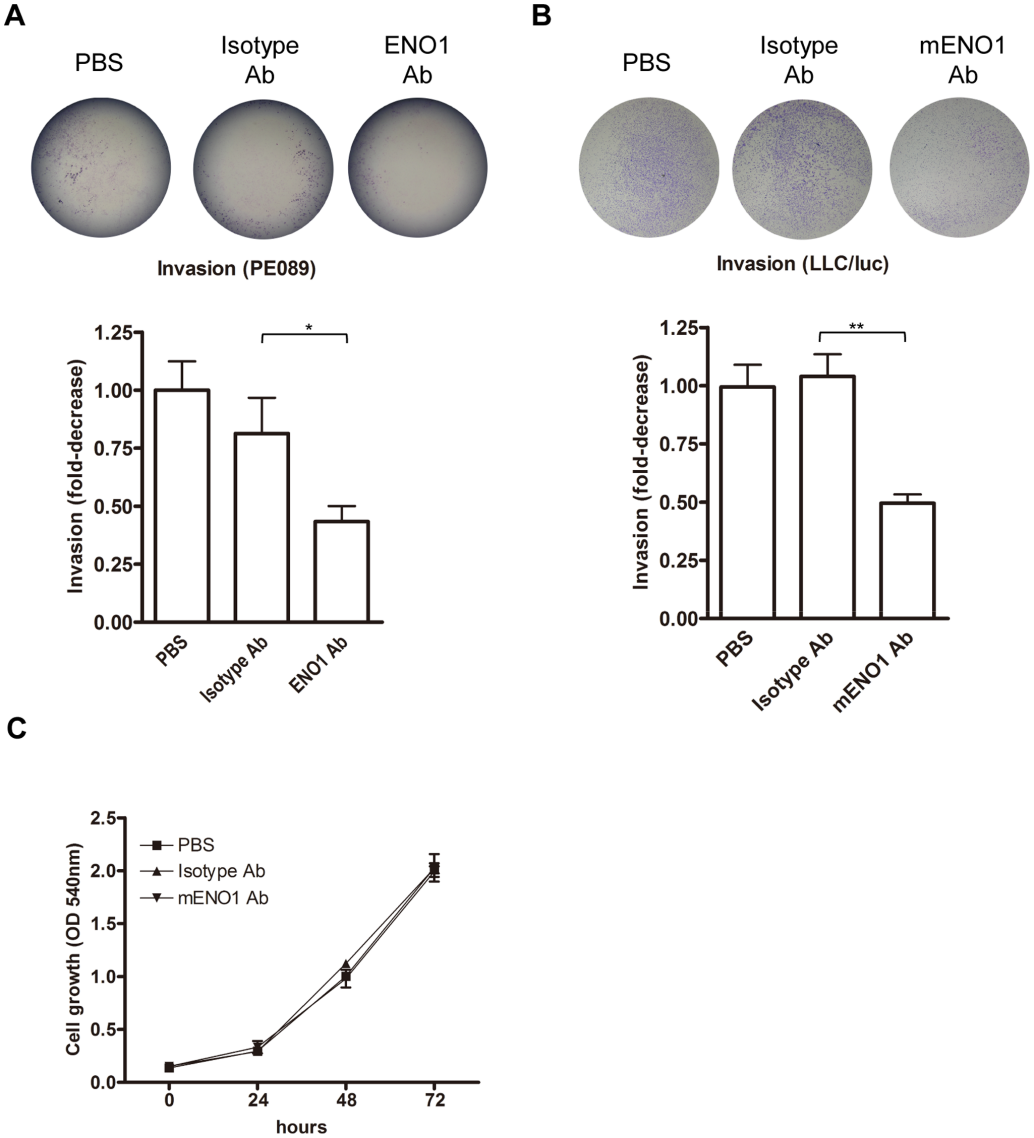
Ab against ENO1 suppressed cell invasion. PE089 (A) or LLC/luc (B) cells were suspended in serum-free medium containing PBS, an ENO1-specific Ab (2 µg/ml), or an isotype-control Ab (2 µg/ml), and then added into transwell chambers consisting of matrigel-coated polycarbonate membranes. The transwell chambers were placed into wells containing medium with 5% FBS as a chemoattractant. After incubation for 24 h, the invading cells on the opposite side of the chambers were determined. (C) The growth of LLC/luc cells in a culture containing PBS, an isotype-control Ab (2 µg/ml), or an Ab against ENO1 (2 µg/ml) was determined by a MTT assay. *p<0.05 and **p<0.01. The error bars were defined as mean±SD.

As MMP2 and 9, substrates in the plasmin proteolytic cascade [34], [35], are involved in the degradation of ECM and invasion of tumor cells [36], the effect of blocking ENO1 on the activation and activity of MMP2/9 was next examined. We observed a low MMP2/9 activity in the culture supernatant of these tumor cells with gelatin zymographic gel analysis (data not shown). Using a more sensitive assay, we observed that culturing with an ENO1-specific Ab significantly reduced the degradation of a fluorescence-labeled gelatin by LLC/luc cells (**Figure 2E**). In vitro transwell assay revealed that addition of ENO-specific Ab leads to a 40% to 50% reduction in the number of PE089 and LLC/luc cells passing through the matrigel-coated membrane (**Figures 3A and 3B**). These results suggest that surface ENO1 participates in tissue invasion of tumor cells. The in vitro growth curve of the LLC/luc cells cultured with the ENO1-specific Ab was similar to that of cells cultured with the isotype-control Ab within the first 72 h (**Figure 3C** **)**, suggesting that the results described above cannot be simply attributed to interference of the ENO1-specific Ab on the growth of tumor cells.

### Down-regulation of ENO1 Inhibits ECM Degradation and Cell Invasion

**Figure 4:**
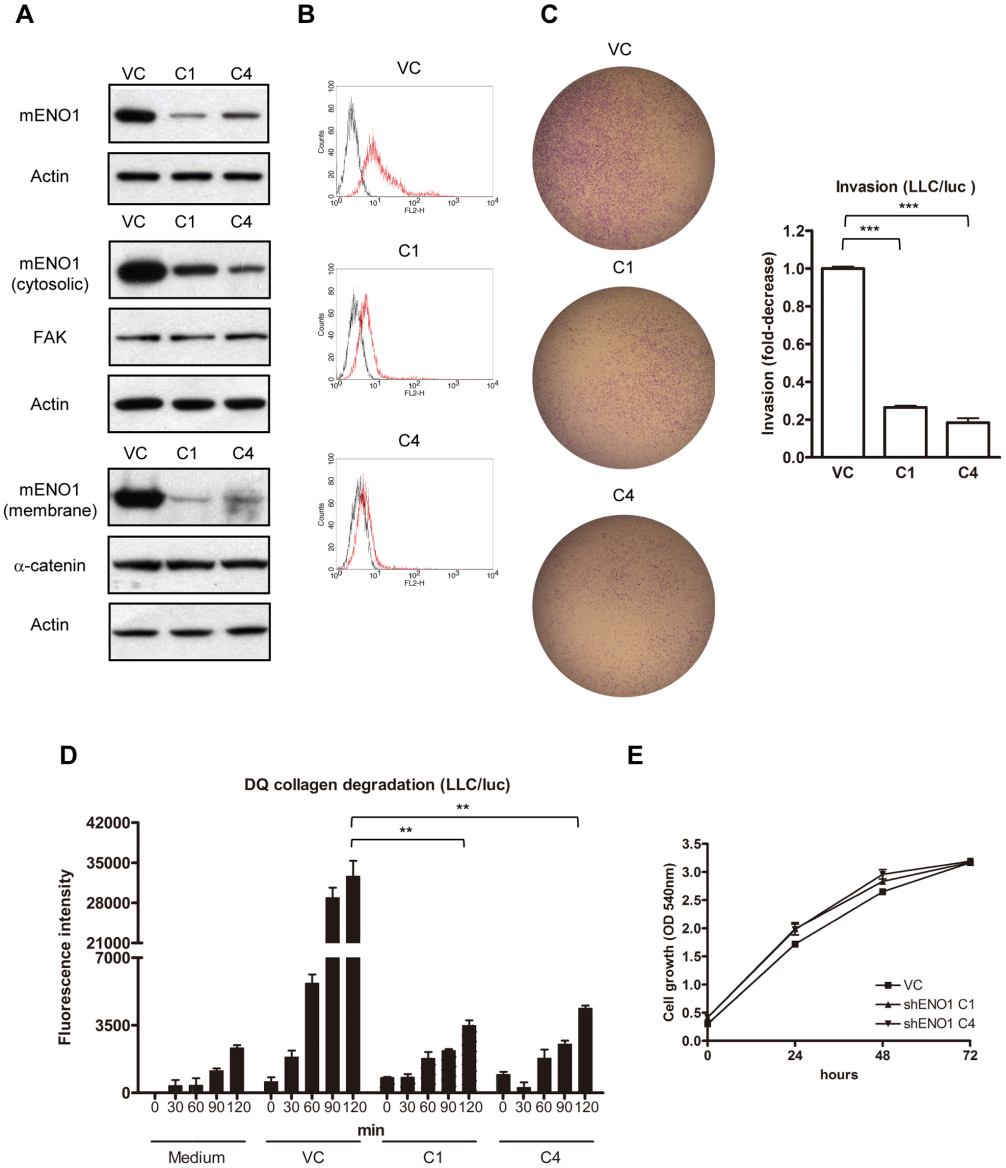
Down-regulation of ENO1 by shRNA reduced collagen degradation and cell invasion. (A) LLC/luc cells were transfected with control (VC) and mENO1-specific (C1 and C4) shRNA plasmids, and the expression of ENO1 was determined by Western blotting analysis. Upper panel: whole cell lysate; middle panel: cytosolic fraction; lower panel: membrane fraction. Expression of actin, FAK, and α-catenin was used as loading controls. (B) Surface expression of mENO1 in various shRNA-transfected LLC/luc cells was further determined by FACS analysis with isotype-control Ab (dark lines) and Ab against mENO1 (red lines). (C) LLC/luc cells transfected with control and mENO1-specific shRNA plasmids were added into transwell chambers consisting of matrigel-coated polycarbonate membranes. The transwell chambers were placed into wells containing medium with 5% FBS as a chemoattractant. After incubation for 24 h, the invading cells on the opposite side of the chambers were determined. (D) The culture supernatant of LLC/luc cells transfected with control and mENO1-specific shRNA plasmids were collected at 30-min intervals and mixed with soluble DQ-collagen for detection of the fluorescence generated after the degradation of DQ-collagen. (E) The growth curve of LLC/luc cells transfected with control and mENO1-specific shRNA plasmids was determined by a MTT assay. **p<0.01 and ***p<0.001. The error bars were defined as mean±SD.

To directly identify the role of ENO1 in ECM degradation and tissue invasion, LLC/luc cells were transfected with ENO1-specific or control shRNA plasmids. Transfection of ENO1-specific shRNA reduced the expression of ENO1 in the cytosol and membrane (**Figures 4A and 4B**), the in vitro invasion capacity (**Figure 4C**), and the extent of DQ-collagen degradation by LLC/luc cells (**Figure 4D**), but had no effect on the cell growth within 72 h (**Figure 4E**). These results link the expression level of ENO1 to the capacity of lung cancer cells to degrade and invade the ECM in vitro. We next examined the role of surface ENO1 on tumor cells in vivo with 4 different animal models.

### Ab against ENO1 Delays Tumor Lung Metastasis in vivo

**Figure 5:**
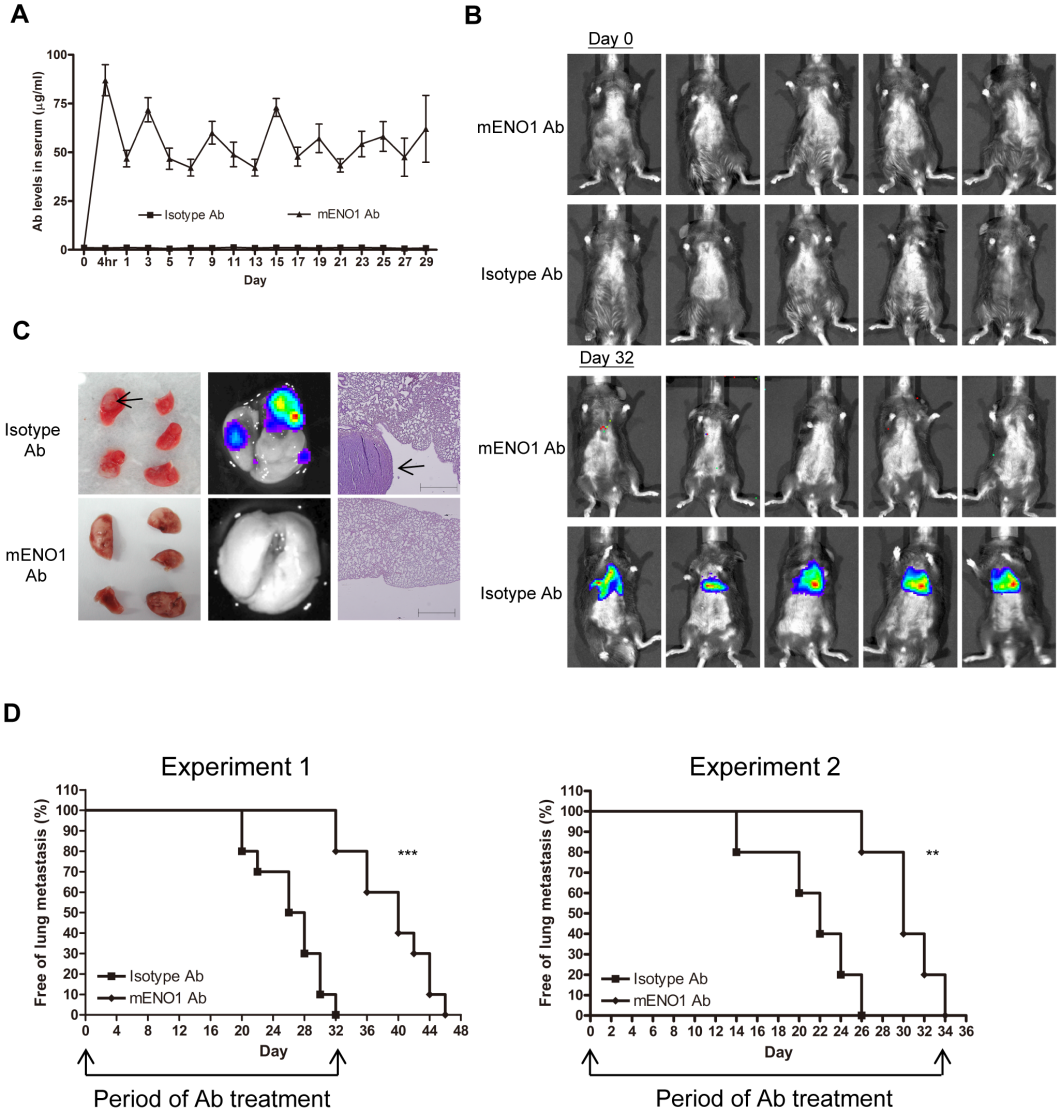
Ab against ENO1 delayed tumor lung metastasis after i.v. implantation of tumor cells. (A) Serum level of Ab against ENO1 in mice adoptively transferred with an isotype-control or mENO1-specific Ab (mENO1 Ab) throughout the experimental period was determined by an ELISA. (B) C57BL/6 mice (n = 10 for experiment 1 and n = 5 for experiment 2) were i.v. injected with LLC/luc cells and adoptively transferred with an isotype-control or mENO1 Ab. Lung metastasis of LLC/luc cells was determined by the presence of luminescence through the IVIS System. Results were obtained from experiment 1 and representative pictures of mice at days 0 and 32 were shown. (C) Macroscopic view of the 5 lobes of lung (left) and the presence of luminescence in the lungs (middle) of two representative mice (experiment 1) treated with an isotype-control (top) and mENO1 Ab (bottom) were shown. Establishment of lung metastasis of LLC/luc cells was confirmed by H&E staining (right). Scale bar = 1mm. The arrows indicated the location of tumor from mice sacrificed on day 32. (D) The percentage of mice without lung metastasis after adoptive transfer of an isotype-control or mENO1 Ab was determined in two independent experiments (experiments 1 and 2). The arrows indicated the period of Ab injection. **p<0.01 and ***p<0.001. The error bars were defined as mean±SD.

In the first model, which was created by i.v. injection of LLC/luc cells into C57BL/6 mice, an equal number of mice were either administered ENO1-specific Ab or an isotype-control Ab during the experimental period. The concentration of ENO1-specific Ab in sera was maintained at a relatively high level (**Figure 5A**). Establishment of lung metastasis was detected using a live-imaging system (**Figure 5B**) and confirmed by pathological examination (**Figure 5C**). Treatment with the ENO1-specific Ab significantly delays the development of lung metastasis in two separate experiments (**Figures 5B and 5D**).

**Figure 6:**
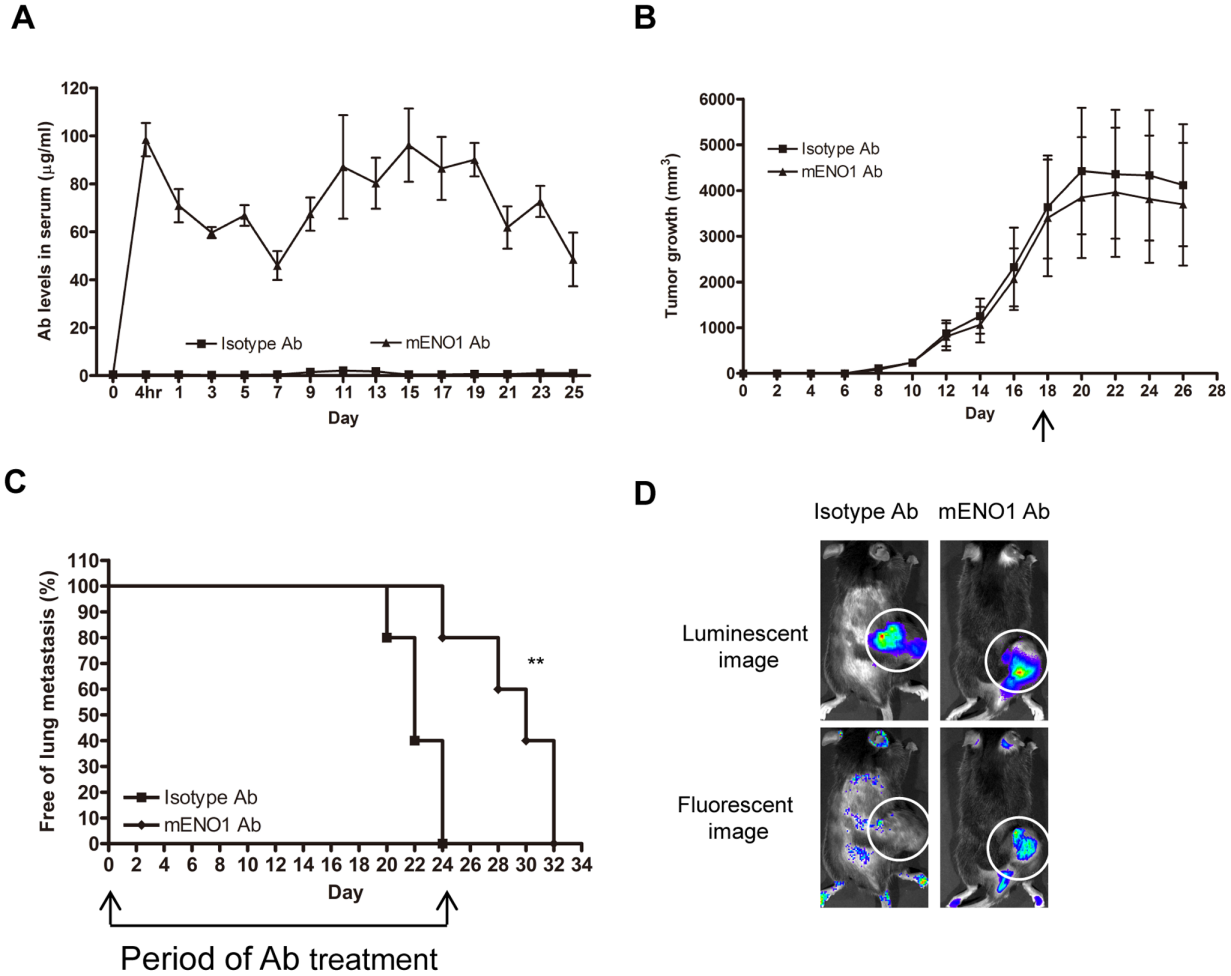
Ab against ENO1 delayed tumor lung metastasis after s.c. implantation of tumor cells. (A) Serum level of Ab against ENO1 in mice adoptively transferred with an isotype-control or mENO1-specific Ab (mENO1 Ab) throughout the experimental period was determined by an ELISA. (B) Tumor growth in mice after s.c. injection of LLC/luc cells and adoptive transfer of an isotype-control Ab or mENO1 Ab was measured every 2 days. To prevent overgrowth of the primary tumor, all mice received 3 Gy of local irradiation on day 18 (indicated by the arrow). (C) The percentage of mice (n = 5) without lung metastasis after adoptive transfer of an isotype-control or mENO1 Ab was determined. The arrows indicated the period of Ab injection. (D) LLC/luc was injected s.c. into C57BL/6 mice. After tumor volume had reached 200 mm^3^, 100 µg Alexa Fluor 488 labeled mENO1 Ab or isotype control Ab (control Ab) was injected i.v. into mice. The fluorescent (indicating Ab) and luminescent (indicating tumor) intensity were detected 4 h later by the IVIS System. The location of tumor was indicated by dotted circle. **p<0.01. The error bars were defined as mean±SD.

In the second animal model, LLC/luc cells were s.c. transplanted into mice and then the mice were treated with either an ENO1-specific or isotype-control Ab as described above for 24 days (**Figure 6A**). Administration of the ENO1-specific Ab did not affect the growth of LLC/luc cells at the s.c. injection site within the first 18 days (**Figure 6B**). The growth of the s.c. tumor of both groups was halted by treatment with 3 Gy of local irradiation at day 18. The development of lung metastasis in mice treated with the ENO1-specific Ab was significantly hindered (**Figure 6C**). To confirm that the administered ENO1-specific Ab was capable of recognizing the s.c. tumor, we labeled the ENO1-specific Ab with Alexa Fluor 488 dye and administered the Ab after the establishment of s.c. LLC/luc tumor. As shown in **Figure 6D**, fluorescence-labeled ENO1-specific Ab accumulated at the s.c. tumor following i.v. injection, indicating that the ENO1-specific Ab can effectively bind to LLC/luc tumor and delay lung metastasis.

### Tumor Bone Metastasis is Suppressed by Systemic Administration of ENO1-specific Ab

**Figure 7:**
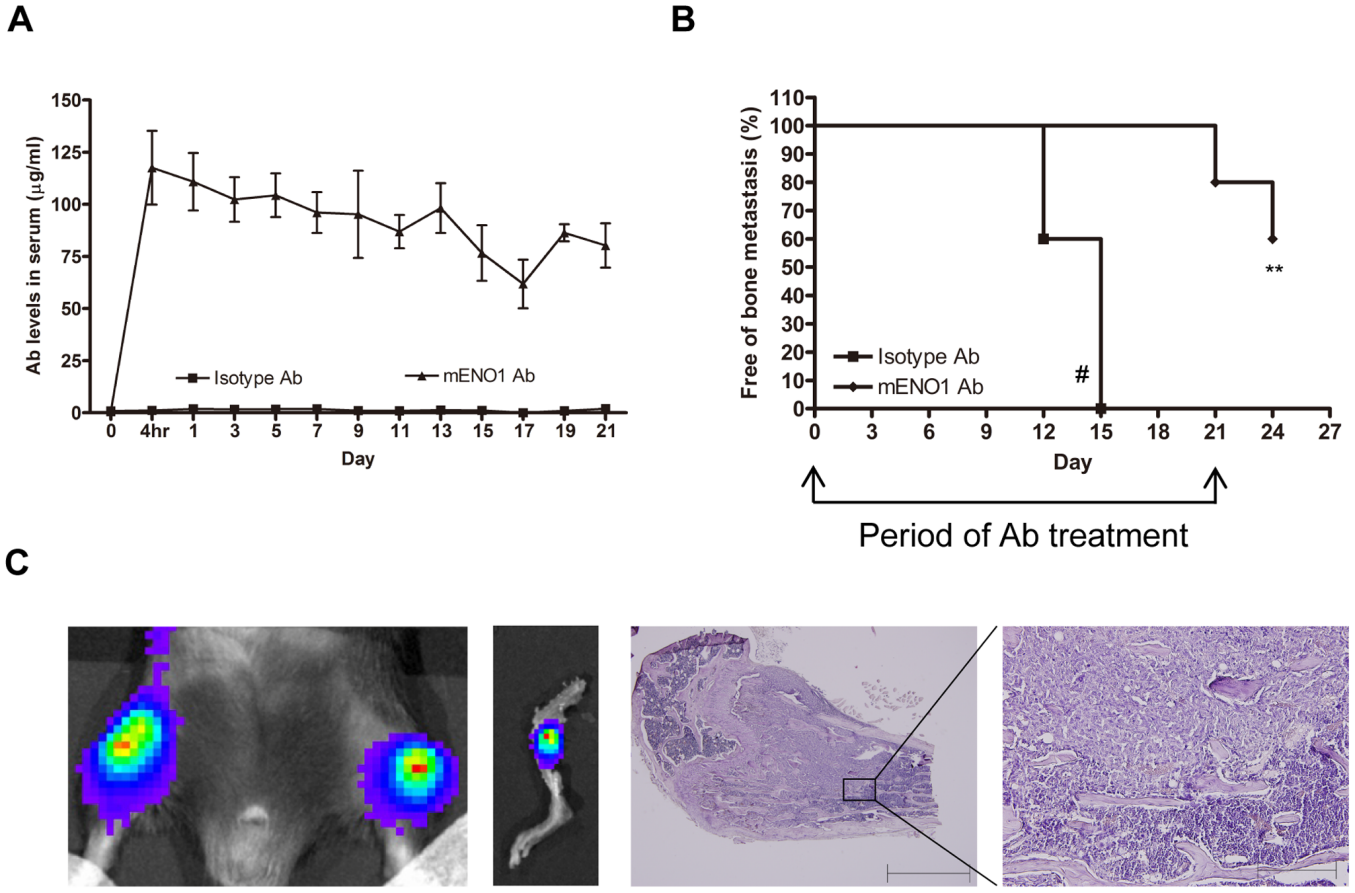
Tumor bone metastasis was suppressed by systemic administration of Ab against ENO1. (A) Serum level of Ab against ENO1 in mice adoptively transferred with an isotype-control or mENO1-specific Ab (mENO1 Ab) during the experimental period was determined by an ELISA. (B) Bone metastasis in mice (n = 5) after intracardiac injection of LLC/luc cells and treatment with an isotype-control or mENO1 Ab was detected by the IVIS System. The percentage of mice remained alive and free of bone metastasis after adoptive transfer of an isotype-control or mENO1 Ab was determined. Two mice treated with the isotype-control Ab died from lung metastasis by day 15 (#). The arrows indicated the period of Ab injection. (C) The tumor in the bone of mice treated with an isotype-control Ab was detected by the IVIS System (left 2 panels). Establishment of bone metastasis was confirmed by H&E staining (in the right 2 panels, scale bar is 1mm and 200 µm in each graph). **p<0.01. The error bars were defined as mean±SD.

In lung cancer patients, the incidence of bone metastasis of cancer cells, a process that is also characterized by a series of ECM degradation and invasion events [33], [37], has been estimated at 30% to 40% [38]. We next investigated the effect of blocking ENO1 on tumor bone metastasis in the third animal model. This model was created by intracardiac injection of LLC/luc BM 2^nd^ cells (see Methods for a description of the development of this cell line) into C57BL/6 mice to allow for formation of bone metastasis, followed by systemic administration of either an ENO1-specific Ab or isotype-control Ab in the same manner as previously described (**Figure 7A**). The mice administered the isotype-control Ab developed lung metastasis between days 6 and 9, whereas those administered the ENO1-specific Ab did so between days 12 and 21 (data not shown). Furthermore, 60% of mice administered the isotype-control Ab developed bone metastasis, as detected by a live-imaging system, with the remainder dying because of lung metastasis at day 15, and all of the mice treated with the ENO1-specific Ab remained free of bone metastasis until day 21 (**Figure 7B**). The establishment of bone metastasis was confirmed by pathological examination (**Figure 7C**).

### Ab against ENO1 Delays Lung Metastasis of Human Cancer Cells in Immune-compromised Mice

**Figure 8:**
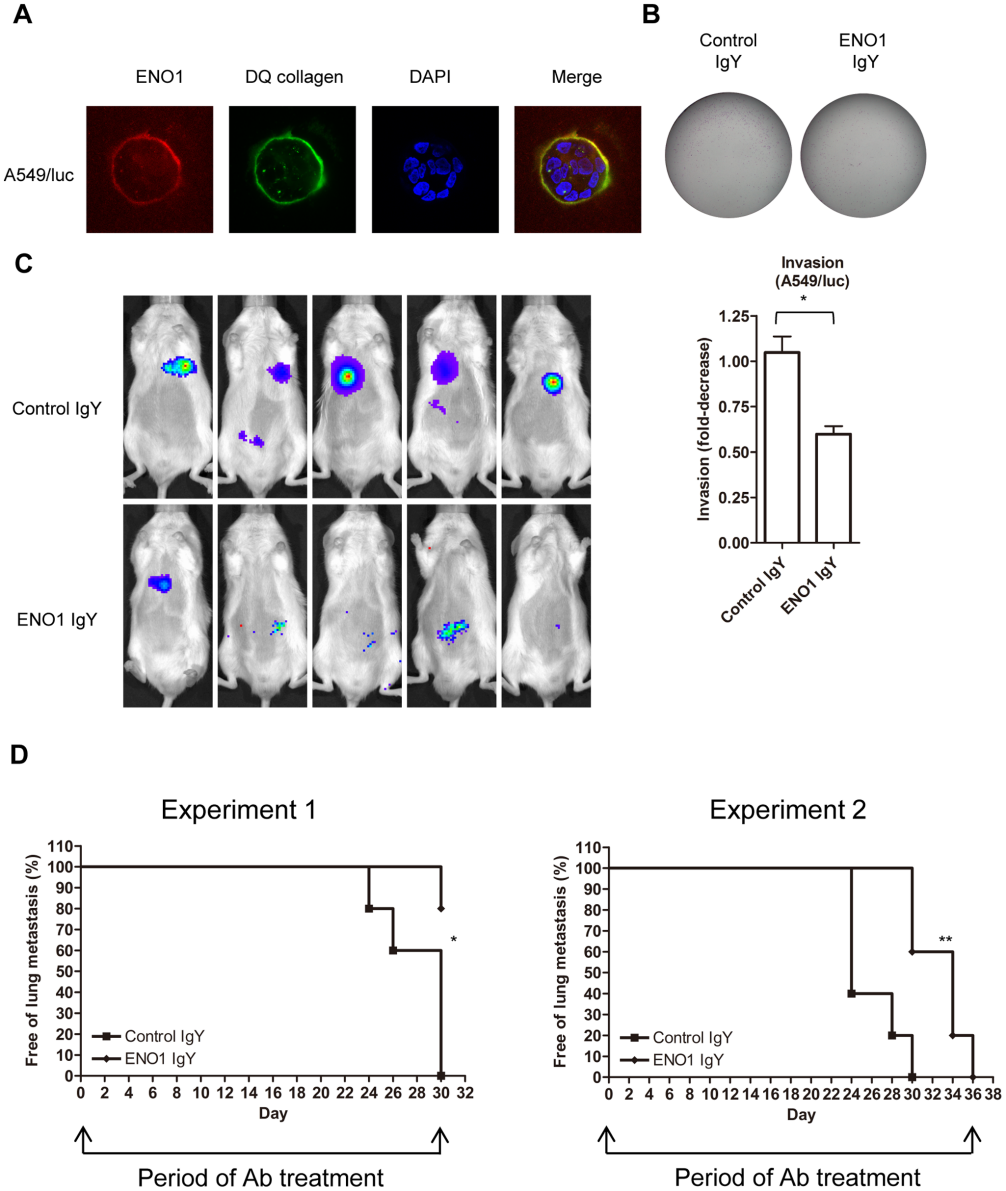
ENO1 accumulated at the pericellular proteolytic site and Ab against ENO1 suppressed invasion and lung metastasis of human A549 lung cancer cells. (A) A549/luc cells were embedded in matrigel containing fluorescence DQ-collagen to allow the growth of cells in a 3D fashion, and then further stained with a PE-labeled Ab against ENO1 to detect the presence of ENO1. The cleaved product of DQ-collagen (green fluorescence) and the presence of ENO1 (red fluorescence) was observed under a confocal microscope. Cells were counterstained with DAPI. (B) In matrigel invasion assay, A549/luc cells in the upper chamber were suspended in serum free medium containing ENO1-specific IgY (ENO1 IgY) or control IgY (10 µg/ml) and medium containing 5% FBS was used as a chemoattractant in the bottom chamber. The number of invaded cells was determined after 24 h. (C) NOD-SCID mice (n = 5 in both experiments 1 and 2) were i.v. injected with A549/luc cells and adoptively transferred with control IgY or ENO1 IgY. Lung metastasis of A549/luc cells was determined by the presence of luminescence through the IVIS System. Results obtained at day 30 of experiment 1 were shown. (D) The percentage of mice without lung metastasis after adoptive transfer of control IgY or ENO1 IgY was determined in two independent experiments (experiments 1 and 2). The arrows indicated the period of Ab injection. *p<0.05. The error bars were defined as mean±SD.

Although we have shown that ENO1-specific Ab has no effect on the growth of LLC/luc cells both in vitro and in vivo under our experimental conditions, we cannot fully rule out the possibility that Ab-mediated immune responses is involved in the inhibition of tumor metastasis. To evaluate this possibility and extend the observation to human lung cancer cells, we conducted the forth animal model using a human lung cancer cell line and the immune-compromised NOD-SCID mice. Because PE089 cells grow very slowly in vivo, we therefore used A549/luc lung cancer cells in this model. As shown in **Figures 8A and 8B**, we confirmed that ENO1 co-localized with DQ collagen degradation in a 3D culture of A549/luc cells and that IgY against human ENO1 significantly suppressed the invasion of A549/luc cells in vitro. Administration of IgY against human ENO1 also effectively suppressed lung metastasis in two separate experiments (**Figures 8C and 8D**).

## Discussion

The cellular function and subcellular localization of ENO1 diverging from its original role in the glycolytic pathway has been reported [39]. In our previous research, we identified ENO1 as a tumor-associated antigen in NSCLC patients and observed its expression on the surface of cancer cells [17]. The expression of ENO1 on the cell surface was also observed in other cancers [21], [22], [40]. Because the coding sequence of ENO1 lacks a transmembrane or a GPI-linkage section, the mechanism by which ENO1 is transported to and displayed on the cell surface and the importance of ENO1 on the surface of tumor cells remains unclear.

Although several surface molecules, including ENO1, integrin, annexin II, and cell surface actin [41], [42], were observed to bind with plasminogen, it is reported that surface-dependent plasminogen activation in activated leukocytes is promoted mainly by ENO1 [41], [43]. High plasminogen expression in tumor cells has been linked to malignancy [10]. The uPA/uPAR system has been reported to mediate tumor metastasis and was proposed to be a potential target of cancer therapy [44], [45]. ENO1 has been observed to co-precipitate with uPAR in a multi-protein complex in ovarian cancer cells [46]. In this study, we demonstrated that surface ENO1 interacted with plasminogen, uPA and uPAR on the PE089 and LLC/luc lung cancer cell lines. It is likely that such a close association between ENO1 and the uPA/uPAR complex may be responsible for the presence of ENO1 on the cell surface. We propose that ENO1 links plasminogen with the uPA/uPAR complex in close proximity on tumor cells that express endogenous plasminogen, uPA, and uPAR, resulting in efficient generation of plasmin and subsequent proteolytic activity on the surface of tumor cells. Indeed, in this study we demonstrated that blocking ENO1 by treatment with an ENO1-specific Ab or suppressing ENO1 expression by treatment with a specific shRNA plasmid reduces plasmin and MMP2/9 activation, ECM degradation, and invasion capacity in lung cancer cells. While adoptive transfer of Ab against ENO1 was observed to suppress the establishment of lung metastasis by lung cancer cells, the prolonged treatment of ENO1-specific Ab at the later stage of metastasis did not significantly improve the therapeutic effect (experiments 1 and 2 in **Figures 5D** **and** **8D**). These results support the role of ENO1 in the early stage of metastasis establishment. Because ENO1-specific Ab did not significantly influence tumor cell growth both in vitro and in vivo in our experimental setting, the effect of ENO1-specific Ab observed in this study is not likely due to Ab-mediated killing of tumor cells. Nevertheless, adoptively transferred ENO1-specific Ab efficiently accumulated at the s.c. site of tumor, suggesting the potential application of delivering therapeutic agents to tumors with ENO1-specific Ab.

One important issue that deserves attention is that posttranslational modification of ENO1 is reported to affect the biological functions of ENO1 [47]. Citrullination, a common feature in inflammation, is a modification of arginine side chains by peptidylarginine deaminase and capable of modifying the structure, function, and antigenicity of proteins [48]. Citrullination of arginine on surface ENO1 is proposed to alter plasminogen binding capacity, leading to decreased fibrinolysis in rheumatoid arthritis [49]. In addition, Ab to ENO1 were detected in sera from patients with early rheumatoid arthritis [50], and Ab against citrullinated ENO1 was reported to play a role in mouse models of arthritis [51]. We have therefore examined the levels of several cytokines involved in inflammation responses in sera of mice administered with ENO1 Ab. We found that there is an increase in several proinflammatory cytokines in mice transplanted with tumor cells in three animal tumor models (**Figure S3**). This is likely resulted from interaction of tumor and host immune cells. Systemic administration of mENO1 Ab didn’t increase and frequently reduce the serum levels of these cytokines. Further experiments revealed that membrane-associated ENO1 from mouse LLC cells was not recognized by Ab against citrulline (**Figure S4**). These results suggest that the observed effect of ENO1 Ab in this study is not likely associated with citrullination of ENO1.

As several types of proMMP are cleaved by plasmin to create active MMP, which continues to catalyze other types of proMMP [52], blocking the plasminogen receptor with a specific Ab inhibits the initial generation of plasmin, which may effectively down regulate the subsequent activation of proMMP [53] and cell invasion [15]. ENO1 is normally expressed in the cytosol and displayed on the cell surface mainly during pathological conditions such as inflammation, autoimmunity, and malignancy [13], [15], [16]. Targeting surface ENO1 may selectively target tumor cells while leaving the majority of normal cells unharmed [40]. We demonstrated that blocking of surface ENO1, which was confirmed to promote ECM degradation and invasion of cancer cells, by treatment with Ab against ENO1 delays tumor lung and bone metastasis in mice. Since surface ENO1 is present in several cancers, these results provide the rationale to develop therapeutic approaches targeting ENO1 in the future.

## Supporting Information

Figure S1 **Expression of ENO1, uPA, uPAR and plasminogen on lung cancer cells.** The expression of ENO1, plasminogen (PLG), uPA, and uPAR on the surface of several common human lung cancer cells and murine LLC cells was determined by flow cytometry.
(PDF)

Figure S2 **Dot-blot analysis demonstrated the effect of ENO1 Ab on the interaction of ENO1, PLG, uPA and uPAR.** After dotting respectively with plasminogen (PLG)(left panel), uPA (middle panel), or uPAR (right panel), together with ENO1 and OVA, the membranes were further incubated with soluble PLG (left), uPA (middle), and uPAR (right) in the presence of the isotype-control Ab, ENO1-specific Ab, control IgY or ENO1 IgY. The binding of dotted ENO1 to soluble PLG, uPA and uPAR were revealed by detecting with Ab against PLG (left), uPA (middle) and uPAR (right), respectively. OVA was used as a blotting control.
(PDF)

Figure S3 **Proinflammatory cytokines in the sera of mice administered with mENO1 Ab.** The levels of several proinflammatory cytokines in the sera of mice transplanted with LLC cells and administered with mENO1 Ab or isotype-control Ab in experiments of three animal tumor models were determined by BD Cytometric Bead Array (CBA). (I): lung metastasis after i.v. injection of tumor cells; (II): lung metastasis after s.c. transplantation of tumor cells; and (III): bone metastasis after intracardiac injection of tumor cells. The levels of cytokines in the culture medium and 24-h culture supernatant of LLC/luc cells were lower than the detection limit of assay (data not shown).
(PDF)

Figure S4 **Western blotting assay of citrullinated protiens in LLC cells.** The whole cell lysate and membrane fraction of LLC cells immunoprecipitated (IP) with anti-mENO1 Ab were immunoblotted (IB) with anti-mENO1 or anti-citrulline Ab. **-**: cell lysate without IP.
(PDF)

Materials and Methods S1 (PDF)
