# Supplementary material for: Surface α-Enolase Promotes Extracellular Matrix Degradation and Tumor Metastasis and Represents a New Therapeutic Target
Source: PLoS One. 2013 Jul 19;8(7):e69354. doi: 10.1371/journal.pone.0069354 (PMC3716638; doi:10.1371/journal.pone.0069354)
Supplement: Figure S1 — Expression of ENO1, uPA, uPAR and plasminogen on lung cancer cells. The expression of ENO1, plasminogen (PLG), uPA, and uPAR on the surface of several common human lung cancer cells and murine LLC cells was determined by flow cytometry. (PDF) [file pone.0069354.s001.pdf]

Figure S1. Expression of ENO1, uPA, uPAR and plasminogen on lung cancer cells.

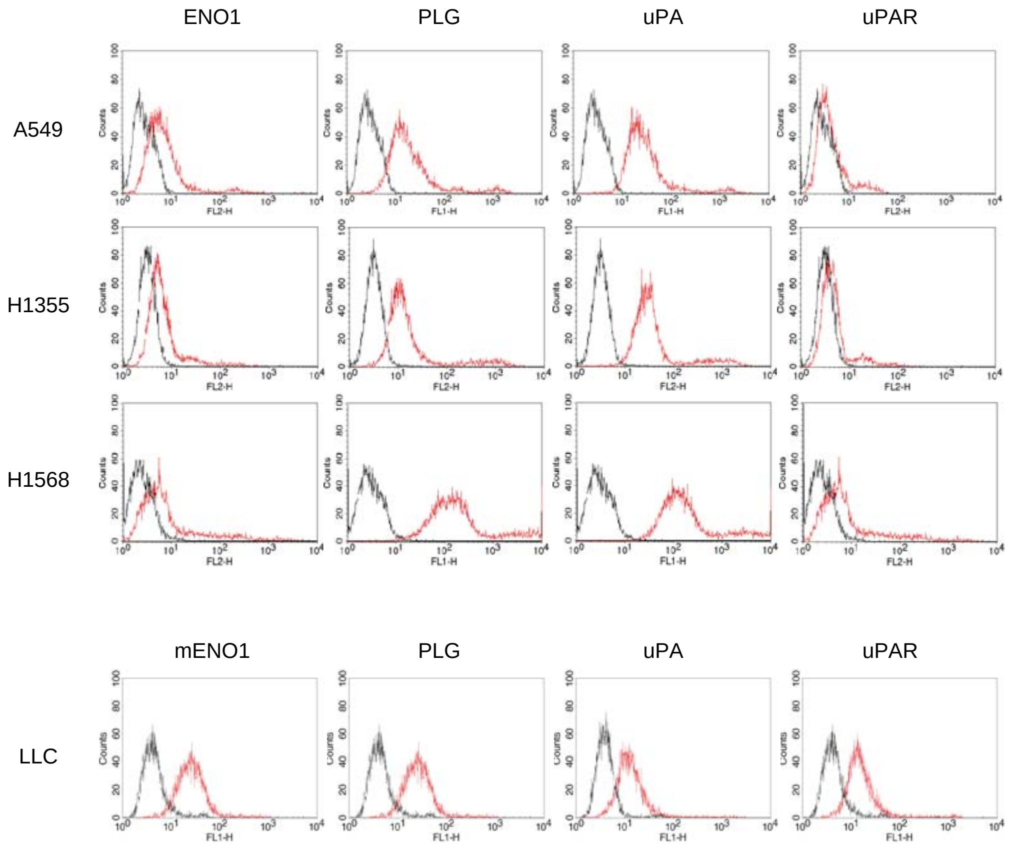

Expression of ENO1, uPA, uPAR and plasminogen on lung cancer cells. The expression of ENO1, plasminogen (PLG), uPA, and uPAR on the surface of several common human lung cancer cells and murine LLC cells was determined by flow cytometry.
