# Supplementary material for: Surface α-Enolase Promotes Extracellular Matrix Degradation and Tumor Metastasis and Represents a New Therapeutic Target
Source: PLoS One. 2013 Jul 19;8(7):e69354. doi: 10.1371/journal.pone.0069354 (PMC3716638; doi:10.1371/journal.pone.0069354)
Supplement: Figure S2 — Dot-blot analysis demonstrated the effect of ENO1 Ab on the interaction of ENO1, PLG, uPA and uPAR. After dotting respectively with plasminogen (PLG)(left panel), uPA (middle panel), or uPAR (right panel), together with ENO1 and OVA, the membranes were further incubated with soluble PLG (left), uPA (middle), and uPAR (right) in the presence of the isotype-control Ab, ENO1-specific Ab, control IgY or ENO1 IgY. The binding of dotted ENO1 to soluble PLG, uPA and uPAR were revealed by detecting with Ab against PLG (left), uPA (middle) and uPAR (right), respectively. OVA was used as a blotting control. (PDF) [file pone.0069354.s002.pdf]

Figure S2. Dot-blot analysis demonstrated the effect of ENO1 Ab on the interaction of ENO1, PLG, uPA and uPAR.

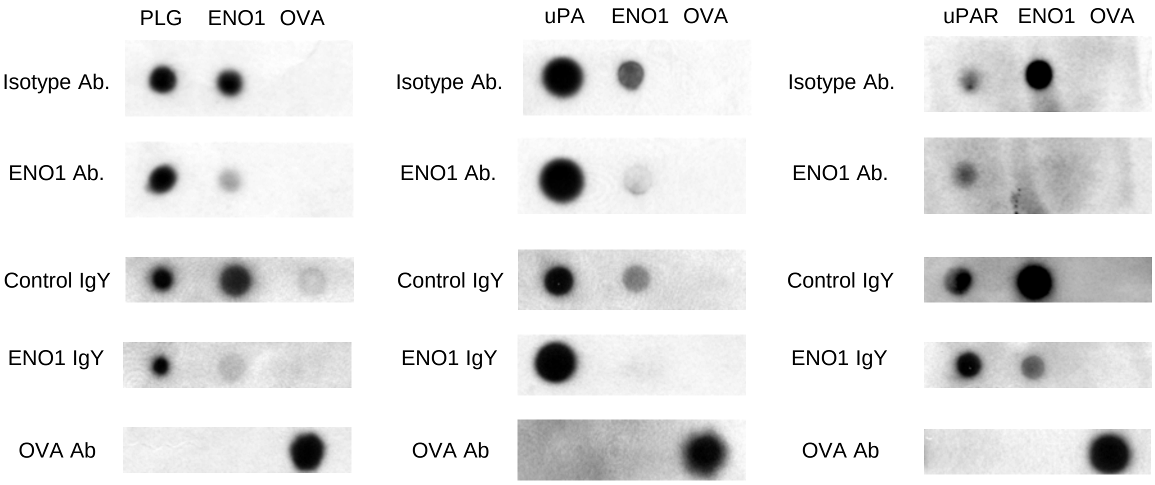

Dot-blot analysis demonstrated the effect of ENO1 Ab on the interaction of ENO1, PLG, uPA and uPAR. After dotting respectively with plasminogen (PLG)(left panel), uPA (middle panel), or uPAR (right panel), together with ENO1 and OVA, the membranes were further incubated with soluble PLG (left), uPA (middle), and uPAR (right) in the presence of the isotype-control Ab, ENO1-specific Ab, control IgY or ENO1 IgY. The binding of dotted ENO1 to soluble PLG, uPA and uPAR were revealed by detecting with Ab against PLG (left), uPA (middle) and uPAR (right), respectively. OVA was used as a blotting control.
