# Supplementary material for: Surface α-Enolase Promotes Extracellular Matrix Degradation and Tumor Metastasis and Represents a New Therapeutic Target
Source: PLoS One. 2013 Jul 19;8(7):e69354. doi: 10.1371/journal.pone.0069354 (PMC3716638; doi:10.1371/journal.pone.0069354)
Supplement: Figure S3 — Proinflammatory cytokines in the sera of mice administered with mENO1 Ab. The levels of several proinflammatory cytokines in the sera of mice transplanted with LLC cells and administered with mENO1 Ab or isotype-control Ab in experiments of three animal tumor models were determined by BD Cytometric Bead Array (CBA). (I): lung metastasis after i.v. injection of tumor cells; (II): lung metastasis after s.c. transplantation of tumor cells; and (III): bone metastasis after intracardiac injection of tumor cells. The levels of cytokines in the culture medium and 24-h culture supernatant of LLC/luc cells were lower than the detection limit of assay (data not shown). (PDF) [file pone.0069354.s003.pdf]

Figure S3. Proinflammatory cytokines in the sera of mice administered with mENO1 Ab.

I. II. III.

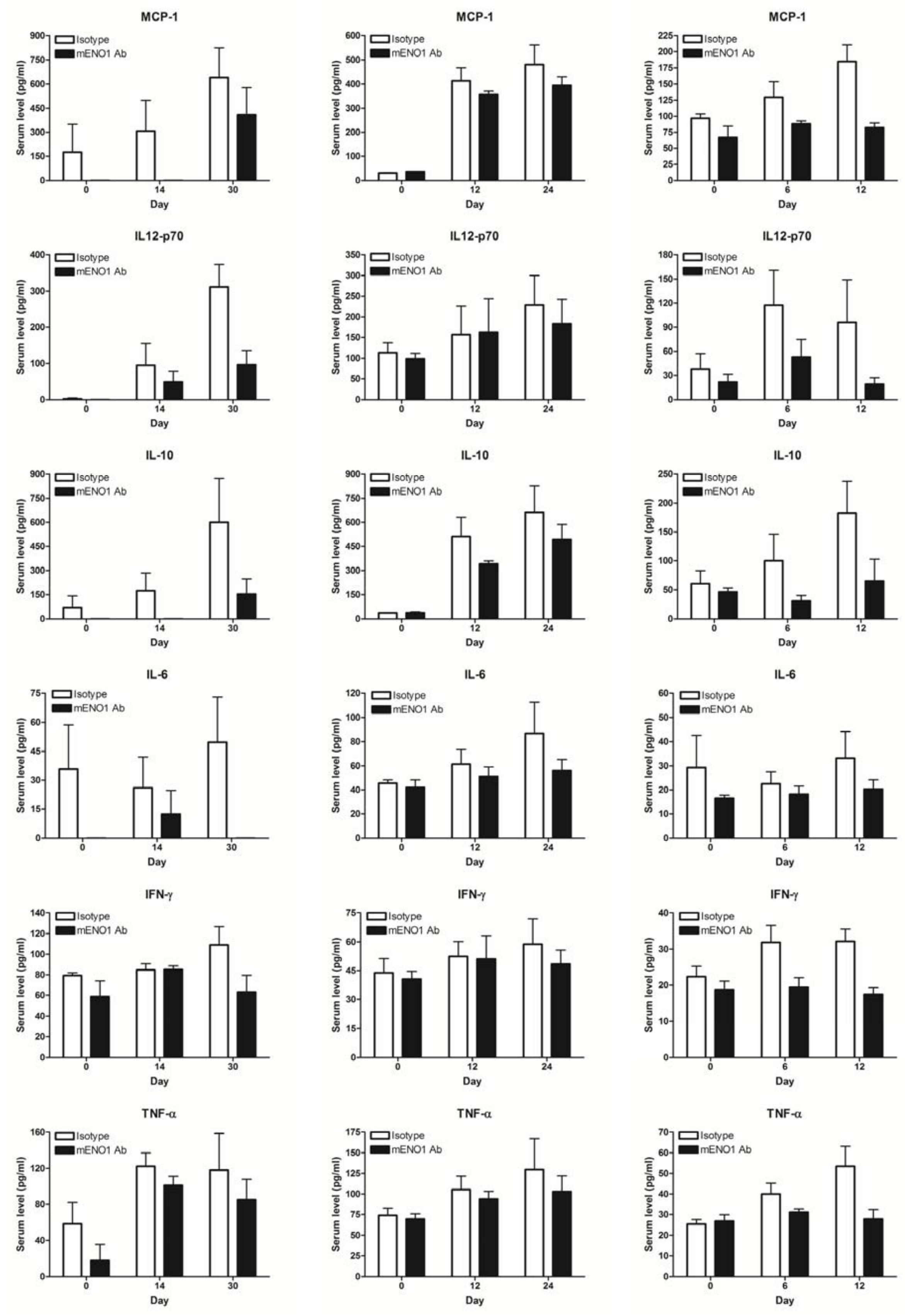

Proinflammatory cytokines in the sera of mice administered with mENO1 Ab. The levels of several proinflammatory cytokines in the sera of mice transplanted with LLC cells and administered with mENO1 Ab or isotype-control Ab in experiments of three animal tumor models were determined by BD Cytometric Bead Array (CBA). (I): lung metastasis after i.v. injection of tumor cells; (II): lung metastasis after s.c. transplantation of tumor cells; and (III): bone metastasis after intracardiac injection of tumor cells. The levels of cytokines in the culture medium and 24-h culture supernatant of LLC/luc cells were lower than the detection limit of assay (data not shown).
