# Supplementary material for: Surface α-Enolase Promotes Extracellular Matrix Degradation and Tumor Metastasis and Represents a New Therapeutic Target
Source: PLoS One. 2013 Jul 19;8(7):e69354. doi: 10.1371/journal.pone.0069354 (PMC3716638; doi:10.1371/journal.pone.0069354)
Supplement: Figure S4 — Western blotting assay of citrullinated protiens in LLC cells. The whole cell lysate and membrane fraction of LLC cells immunoprecipitated (IP) with anti-mENO1 Ab were immunoblotted (IB) with anti-mENO1 or anti-citrulline Ab. -: cell lysate without IP. (PDF) [file pone.0069354.s004.pdf]

Figure S4. Western blotting assay of citrullinated protiens in LLC cells.

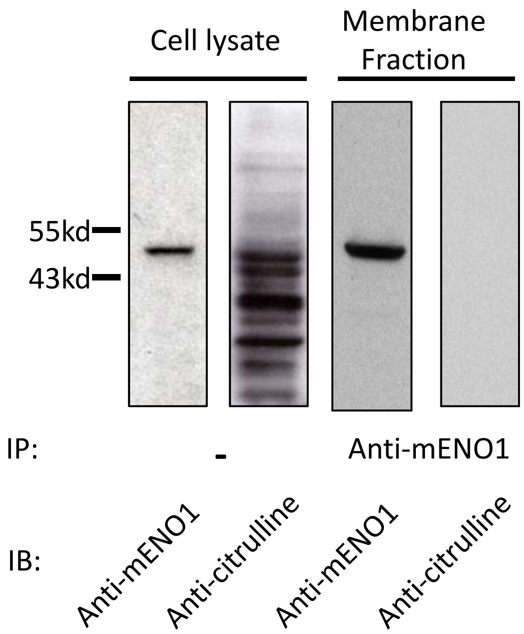

Western blotting assay of citrullinated protiens in LLC cells. The whole cell lysate and membrane fraction of LLC cells immunoprecipitated (IP) with anti-mENO1 Ab were immunoblotted (IB) with anti-mENO1 or anti-citrulline Ab. **-**: cell lysate without IP.
