# Supplementary material for: Surface α-Enolase Promotes Extracellular Matrix Degradation and Tumor Metastasis and Represents a New Therapeutic Target
Source: PLoS One. 2013 Jul 19;8(7):e69354. doi: 10.1371/journal.pone.0069354 (PMC3716638; doi:10.1371/journal.pone.0069354)
Supplement: Materials and Methods S1 — (PDF) [file pone.0069354.s005.pdf]

Supplementary materials and methods:

Cell lines. The murine LL/2 Lewis lung carcinoma cell line (LLC) and human lung cancer cell lines A549, H1355 and H1568 were obtained from American Type Culture Collection (Manassas, VA).

Ab. The mouse anti-uPA Ab was purchased from Millipore. The mouse anti-uPAR Ab was purchased from R&D system (Minneapolis, MN). The goat anti-plasminogen Ab was purchased from ICN Immunobiologicals (Costa Mesa, CA). The rabbit anti-citrulline Ab was purchased from Abcam. Rabbit polyclonal anti-ENO1 Ab and rabbit isotype-control Ab were purchased from GeneTex. HRP-conjugated goat anti-mouse IgG, HRP-conjugated donkey anti-goat IgG and HRP-conjugated goat anti-rabbit IgG Ab were obtained from Santa Cruz Biotechnology.

Flow cytometric analysis. The flow cytometric analysis of surface staining of ENO1, plasminogen, uPA, and uPAR in human lung cancers and LLC cells was performed as described in the Materials and Methods section.

Dot blotting assay. To analyze the effect of ENO1-specific Ab on the binding of ENO1 to plasminogen, uPA, and uPAR, 1 µg of plasminogen, uPA, or uPAR (respectively), together with ENO1 and OVA, were dotted on a strip of nitrocellulose membrane. After blocking with 5% BSA/PBS for 30 min at room temperature, the membranes were incubated respectively with 1 µg/ml of soluble plasminogen, uPA, and uPAR in PBS containing 1 µg/ml rabbit isotype-control Ab, rabbit anti-ENO1 Ab, ENO1 IgY or control IgY for 1 h at room temperature. The membranes were washed with PBS containing 0.1% Tween 20. After hybridization with the goat anti-plasminogen, mouse anti-uPA, mouse anti-uPAR Ab or mouse anti-OVA Ab respectively, in 1% BSA at a dilution of 1:1000, the membranes were incubated with HRP-conjugated anti-goat or anti-mouse IgG Ab. The signals from the membranes were then visualized using the ECL system.

Detection of proinflammatory cytokines. The levels of several proinflammatory cytokines in culture medium, 24-h culture supernatant of LLC/luc cells ($1x10^6$ cells in 10 ml culture medium), and mouse sera (collected at different time points after tumor transplantation) were determined following the manufactures' protocol of a CBA Mouse Inflammation Kit (Becton Dickinson). The limit of detection in CBA: interleukin-6 (IL-6), 5 pg/ml; interleukin-10 (IL-10), 17.5 pg/ml; monocyte chemoattractant protein-1 (MCP-1), 52.7 pg/ml; interferon-γ (IFN-γ), 2.5 pg/ml; Tumor necrosis factor-α (TNF-α), 7.3 pg/ml; interleukin-12p70 (IL-12p70), 10.7 pg/ml.

Western blotting assay of citrullinated protein. Membrane fraction from LLC/luc cells was obtained following the manufactures' protocol of the ProteoExtract® Native Membrane Protein Extraction kit (Millipore). The whole cell lysate and proteins co-immunoprecipitated with anti-ENO1 Ab were detected with anti-ENO1 Ab or rabbit anti-citrulline Ab, followed by HRP-conjugated goat anti-mouse IgG Ab or HRP-conjugated goat anti-rabbit IgG Ab, respectively. The signals from the membranes were then visualized using the ECL system.
